# Rampant chloroplast capture in Sarracenia revealed by plastome phylogeny

**DOI:** 10.3389/fpls.2023.1237749

**Published:** 2023-08-14

**Authors:** Ethan Baldwin, Mason McNair, Jim Leebens-Mack

**Affiliations:** ^1^ Department of Plant Biology, University of Georgia, Athens, GA, United States; ^2^ Department of Plant & Environmental Science, Clemson University, Florence, SC, United States

**Keywords:** hybridization, chloroplast capture, gene flow, carnivorous plant, *Sarracenia*, phylogenomics, plastome

## Abstract

Introgression can produce novel genetic variation in organisms that hybridize. Sympatric species pairs in the carnivorous plant genus *Sarracenia* L. frequently hybridize, and all known hybrids are fertile. Despite being a desirable system for studying the evolutionary consequences of hybridization, the extent to which introgression occurs in the genus is limited to a few species in only two field sites. Previous phylogenomic analysis of *Sarracenia* estimated a highly resolved species tree from 199 nuclear genes, but revealed a plastid genome that is highly discordant with the species tree. Such cytonuclear discordance could be caused by chloroplast introgression (i.e. chloroplast capture) or incomplete lineage sorting (ILS). To better understand the extent to which introgression is occurring in *Sarracenia*, the chloroplast capture and ILS hypotheses were formally evaluated. Plastomes were assembled *de-novo* from sequencing reads generated from 17 individuals in addition to reads obtained from the previous study. Assemblies of 14 whole plastomes were generated and annotated, and the remaining fragmented assemblies were scaffolded to these whole-plastome assemblies. Coding sequence from 79 homologous genes were aligned and concatenated for maximum-likelihood phylogeny estimation. The plastome tree is extremely discordant with the published species tree. Plastome trees were simulated under the coalescent and tree distance from the species tree was calculated to generate a null distribution of discordance that is expected under ILS alone. A t-test rejected the null hypothesis that ILS could cause the level of discordance seen in the plastome tree, suggesting that chloroplast capture must be invoked to explain the discordance. Due to the extreme level of discordance in the plastome tree, it is likely that chloroplast capture has been common in the evolutionary history of *Sarracenia*.

## 1 Introduction

Evolutionary biologists have long been interested in hybridization as a process that generates biodiversity. Hybridization leading to introgression introduces genetic information to a species, which increases genetic variation for selection to act on and provides opportunity for adaptive evolution (Pease et al., 2016; Grant and Grant, 2019; Meier et al., 2019). Organisms that readily hybridize may be subject to these evolutionary forces. However, the formation of hybrids does not imply that introgression (transfer of genome segments between hybridizing species) is occurring, as hybrids must reproduce with the parental population and introgressed alleles must survive in the face of natural selection and genetic drift. Identifying the extent to which hybridizing taxa are exchanging genetic material sheds light on the processes that generate and maintain variation within them.

*Sarracenia* L. is a genus of 8-11 species of carnivorous plants native to North America. It is one of the three extant genera in the family Sarraceniaceae, with species forming tube shaped traps adapted to catch and digest insects. Due to this unique adaptation they are commonly called pitcher plants, although pitcher-shaped carnivorous leaves have evolved convergently in at least two other lineages (*Nepenthes* L., *Cephalotus* Labill.) (Albert et al., 1992). Most *Sarracenia* species occur sympatrically with at least one other species, and all species pairs can produce fertile hybrids (Bell, 1952). Hybrids between sympatric species are frequently observed in nature (Bell, 1952), and population genetics studies using a few microsatellite loci have shown evidence of gene-flow between species at some sites and not at others (Furches et al., 2013; Rentsch and Holland, 2020). The forces maintaining species boundaries are not well known, but it is possible that outbreeding depression is contributing to species coherence in the face of hybridization. *Sarracenia* hybrids exhibit intermediate pitcher morphology which may decrease prey capture efficacy. Another possible factor contributing to the maintenance of species boundaries is asynchronous flowering phenology (Bell, 1952).

*Sarracenia* diverged from the rest of Sarraceniaceae an estimated 23 MYA, with most of the diversification within *Sarracenia* occurring between 1-3 MYA (Ellison et al., 2012). Given the rapid speciation, significant gene tree discordance is expected due to incomplete lineage sorting (ILS) (Degnan and Rosenberg, 2009). Despite this, Stephens et al. (2015) estimated a multi-species coalescent phylogeny using 199 nuclear genes that resolved most of the species relationships with high support. This study also presented a plastome tree that was highly discordant with the nuclear tree; no species was reciprocally monophyletic. Cytonuclear discordance such as this can be the result of ILS or introgression of the plastid genome, otherwise referred to as chloroplast capture.

Although the plastome phylogeny estimated in Stephens et al. (2015) is relatively well supported, the analysis was limited by the recovery of only 42kbp of plastome sequence limited to the long single copy and short single copy regions of the plastome. To confirm that the extreme cytonuclear discordance observed in the Stephens et al. (2015) phylogenies was not an artifact of a lack of data, we reassembled plastomes from those sequencing reads using an alternative assembly pipeline to recover more sequence. Seventeen additional accessions are added to this analysis. The cause of cytonuclear discordance is formally assessed using a coalescent based simulation approach to distinguish between ILS and chloroplast capture. Additionally, whole plastomes are assembled and gene content evolution is assessed within the context of carnivory.

## 2 Materials and methods

### 2.1 Sequence data

Leaf tissue was obtained from 17 individuals in total: 11 accessions were obtained from the Atlanta Botanical Garden’s living conservation collection (*S. oreophila*, *S. jonesii*, *S. alata*, *S. alabamensis* and *S. rubra*) and six accessions were obtained from two field sites (*S. rubra subsp. rubra* and *S. rubra subsp. viatorum*). DNA was extracted from silica dried samples using the Qiagen DNeasy Plant Mini Kit. Library prep was performed using the Kapa Biosystems HyperPlus Kit using iTru adapters (Glenn et al., 2019). Libraries were pooled at equal concentrations and enriched for putative single-copy orthologs enrichment using the Angiosperms353 bait set (Johnson et al., 2019). The enriched pool was sequenced on an Illumina NextSeq 500 at the Georgia Genomics and Bioinformatics Core using a High Output 300 cycle flow cell generating 150bp paired-end reads.

In addition, sequencing reads from Stephens et al. (2015) were downloaded from NCBI Short Read Archive. The Stephens et al. data set includes 71 accessions of *Sarracenia* and 4 accessions of outgroups in Sarraceniaceae (*Heliamphora minor* and *Darlingtonia californica*).

### 2.2 Plastome assembly

All raw reads were trimmed using Trimmomatic (v. 0.39) (Bolger et al., 2014). Both the new data set and the data set obtained from Stephens et al. were sequenced from libraries enriched for targeted nuclear loci. However, the majority of the reads from both data sets are off-target. Stephens et al. reported an average of 1.6% of reads on target, and analysis of the new data set revealed that less than 1% of the reads were on target. The large proportion of off-target reads enable the assembly of the plastome.

Initial *de-novo* plastome assembly was attempted with GetOrganelle (v. 1.7.5.2) (Jin et al., 2020). GetOrganelle often produced two assembly versions differing only in the orientation of the short single copy regions (SSC). SSC orientation was determined by aligning assemblies to the reference plastome (*Clethra* L. *delavayi*, Genbank accession NC_041129) using MUMmer (v. 4.0.0) (Kurtz et al., 2004), and only the assemblies with concordant SSC orientation were retained.

GetOrganelle did not generate complete *de-novo* plastome assemblies from every sample. In these cases, the following reference-based pipeline was used. Reads were aligned to one of the complete *Sarracenia* plastome assemblies using BWA (v. 0.7.17) (Li et al., 2009). The aligned reads were then extracted and assembled *de-novo* using SPAdes (Bankevich et al., 2012). Afin (https://github.com/afinit/afin) was used to extend the resulting contigs and fuse any contigs with significant overlap. At this stage, assemblies were either mostly complete (1-3 contigs consisting of the large single copy region (LSC), short single copy regions (SSC), and one IR), or they were more fragmented. The mostly complete assemblies were manually pasted together. The IR boundaries were verified by mapping reads to the assemblies and identifying the coordinate where half of the reads spanned the IR and LSC and the other half spanned the IR and SSC.

### 2.3 Plastome annotation

Complete plastome assemblies were annotated using PGA (Qu et al., 2019). Fragmented assemblies were aligned to one of the complete, PGA annotated plastomes using the Minimap2 (v. 2.17) (Li, 2018) plugin in Geneious. The “transfer annotation” function was used before generating a consensus sequence.

### 2.4 Alignment and phylogeny estimation

Coding sequences (CDS) from 79 plastid genes were extracted from the annotated assemblies and aligned with MAFFT (v. 7.470) (Katoh and Standley, 2013). All resulting gene alignments were concatenated. Regions of the concatenated alignment that were poorly aligned or had gaps in 50% or more of the samples were filtered out of the gene alignments using Gblocks (v. 0.91b) (Castresana, 2000). A maximum-likelihood phylogeny was estimated from the concatenated gene alignments using IQ-Tree (v. 2.0.6) (Nguyen et al., 2015). 1000 bootstrap replicates were performed using UFBoot (Minh et al., 2013). The GTR + F + R4 substitution model was used.

### 2.5 Plastome tree simulations

To differentiate between incomplete lineage sorting (ILS) and chloroplast capture, a tree simulation approach similar to Folk et al., 2017 (Folk et al., 2016) was used. Plastome trees under ILS were simulated using the dendropy python package (v. 4.5.2) (Sukumaran and Holder, 2010) with the species tree from Stephens et al. (2015) as a guide tree. Since plastomes are effectively haploid and inherited uniparentally, plastomes have one quarter of the effective population size of diploid nuclear loci. Since the guide tree used for these simulations was estimated exclusively using nuclear loci, its branch lengths were scaled by four to account for the effective population size differential between plastomes and nuclear loci. A distribution of tree discordance under the null hypothesis of ILS was generated by calculating a tree distance metric [information-based generalized Robinson-Foulds distance (Smith, 2020)] between 1000 simulated trees and the species tree. Then the distance between the empirical plastome tree from this study and the species tree was calculated and compared to the null distribution. Since the empirical plastome tree has samples that are not in the Stephens et al. (2015) species tree, those tips were dropped from the plastome tree to enable calculating distance.

## 3 Results

### 3.1 Plastome assemblies

Fourteen complete, circularized plastomes have been assembled and annotated including the following *Sarracenia* species: *S. jonesii*, *S. alabamensis*, *S. oreophila*, *S. rubra subsp. gulfensis, S. rubra subsp. rubra*, and *S. rubra subsp. viatorum*. Average assembly statistics for the all assemblies are shown in **Table 1**. The assembly pipeline for fragmented assemblies recovered an average of 114kbp of plastome sequence, almost tripling the 42kbp recovered in Stephens et al. (2015). The use of different references is one potential factor explaining this difference; this study used a complete *Sarracenia* plastome (Ericales) as a reference whereas Stephens et al. (2015) used a plastome from *Vitis vinifera* (Vitales). Eighty protein-coding genes were extracted from assemblies, and sequences were aligned for all samples, and alignments were concatenated for the phylogeny estimation.

**Table 1:** Accession information and assembly statistics for all samples used in this study.

Taxon	Sample ID	NCBI Biosample	Collector	Herbarium ID	Total contigs	Total length
*Darlingtonia californica*						
	DarlingtoniaOR_j028	SAMN03354578	J. D. Stephens	UGA66	36	121605
	DarlingtoniaUN1_j029	SAMN03354579	J. D. Stephens	N/A	65	116346
	DarlingtoniaUN2_j030	SAMN03354580	J. D. Stephens	UGA54	60	112573
*Heliamphora minor*						
	HeliamphoraVE_j031	SAMN03354581	J. D. Stephens	UGA55	45	130627
*S. alabamensis*						
	AlabamensisAL_j018	SAMN03354582	J. D. Stephens	UGA19	76	110275
	Alabamensis_m004	SAMN31020169	E. Baldwin	1004	1	154984
*S. alata*						
	AlataMS1_j033	SAMN03354583	J. D. Stephens	UGA21	51	118063
	AlataMS2_j034	SAMN03354584	J. D. Stephens	N/A	62	117941
	AlataLA1_j035	SAMN03354585	J. D. Stephens	UGA67	25	125730
	AlataTX_j036	SAMN03354586	J. D. Stephens	TAES253951	39	123698
	AlataLA2_j037	SAMN03354587	J. D. Stephens	UGA60	38	126597
	Alata_m003	SAMN36416359	E. Baldwin	1003	29	149266
*S. flava*						
	FlavaGA_j039	SAMN03354588	J. D. Stephens	UGA15	57	115895
	FlavaFL_j042	SAMN03354589	J. D. Stephens	UGA65	1	153868
	FlavaNC1_j045	SAMN03354590	J. D. Stephens	UGA48	34	125019
	FlavaSC_j046	SAMN03354591	J. D. Stephens	UGA45	18	129423
	FlavaNC2_j047	SAMN03354592	J. D. Stephens	UGA50	22	129654
	FlavaVA_j048	SAMN03354593	J. D. Stephens	UGA64	23	128338
*S. flava var. rubricorpora*						
	FlavaRubricorpaFL1_j041	SAMN03354594	J. D. Stephens	UGA18	14	132800
	FlavaRubricorpaFL2_j043	SAMN03354595	J. D. Stephens	UGA18	15	128594
*S. flava var. rugelii*						
	FlavaRugeliiGA1_j038	SAMN03354597	J. D. Stephens	UGA26	52	117830
	FlavaRugeliiGA2_j040	SAMN03354598	J. D. Stephens	UGA44	26	126308
	FlavaRugeliiAL_j044	SAMN03354596	J. D. Stephens	UGA51	8	130660
*S. jonesii*						
	JonesiiSC1_j023	SAMN03354599	J. D. Stephens	UGA32	24	125346
	JonesiiNC1_j024	SAMN03354600	J. D. Stephens	UGA31	9	127465
	JonesiiNC2_j025	SAMN03354601	J. D. Stephens	UGA33	66	116650
	JonesiiSC2_j026	SAMN03354602	J. D. Stephens	UGA30	53	118729
	JonesiiNC1_m007	SAMN31020170	E. Baldwin	1007	1	151409
	JonesiiSC1_m008	SAMN31020171	E. Baldwin	1008	1	151385
*S. minor*						
	MinorGA1_j056	SAMN03354609	J. D. Stephens	N/A	23	126435
	MinorGA2_j058	SAMN03354610	J. D. Stephens	UGA8	82	111289
	MinorGA3_j059	SAMN03354611	J. D. Stephens	UGA39	57	117630
	MinorSC1_j060	SAMN03354612	J. D. Stephens	UGA46	20	127646
	MinorSC2_j062	SAMN03354613	J. D. Stephens	UGA13	32	124716
*S. minor var. okefenokeensis*						
	MinorOkefenokeensisGA_j055	SAMN03354614	J. D. Stephens	UGA23	22	125047
*S. oreophila*						
	OreophilaAL1_j063	SAMN03354615	J. D. Stephens	UGA2	9	128216
	OreophilaAL2_j064	SAMN03354616	J. D. Stephens	UGA28	32	126491
	OreophilaAL3_j065	SAMN03354617	J. D. Stephens	UGA27	66	95604
	OreophilaNC_j066	SAMN03354618	J. D. Stephens	UGA20	29	124312
	OreophilaAL4_j067	SAMN03354619	J. D. Stephens	UGA24	40	120414
	OreophilaGA_j068	SAMN03354620	J. D. Stephens	UGA22	43	118692
	Oreophila_m002	SAMN31020172	E. Baldwin	1002	1	156118
*S. psittacina*						
	PsittacinaGA1_j070	SAMN03354621	J. D. Stephens	UGA43	40	121580
	PsittacinaAL1_j072	SAMN03354623	J. D. Stephens	UGA11	38	128169
	PsittacinaGA3_j073	SAMN03354624	J. D. Stephens	UGA10	23	129214
	PsittacinaAL2_j074	SAMN03354625	J. D. Stephens	UGA1	43	122414
	PsittacinaFL_j075	SAMN03354626	J. D. Stephens	UGA35	36	119271
	PsittacinaAL3_j076	SAMN03354627	J. D. Stephens	UGA53	18	128493
	PsittacinaLA_j077	SAMN03354628	J. D. Stephens	UGA59	29	126403
*S. purpurea ssp. purpurea*						
	PurpureaPurpureaNS_j006	SAMN03354629	J. D. Stephens	UGA61	37	124790
	PurpureaPurpureaWI1_j007	SAMN03354630	J. D. Stephens	UGA47	39	119984
	PurpureaPurpureaWI2_j008	SAMN03354631	J. D. Stephens	UGA47	56	125476
*S. purpurea ssp. venosa*						
	PurpureaVenosaGA_j001	SAMN03354463	J. D. Stephens	UGA12	28	126463
	PurpureaVenosaNC_j003	SAMN03354632	J. D. Stephens	UGA49	33	124039
	PurpureaVenosaMD_j004	SAMN03354633	J. D. Stephens	UGA62	61	121892
	PurpureaVenosaVA_j005	SAMN03354634	J. D. Stephens	UGA63	48	123570
*S. purpurea ssp. venosa var. montana*						
	PurpureaMontanaGA_j078	SAMN03354636	J. D. Stephens	UGA41	31	126220
	PurpureaMontanaNC_j079	SAMN03354635	J. D. Stephens	UGA34	39	118268
*S. rosea (S. purpurea ssp. venosa var. burkii)*						
	RoseaFL2_j002	SAMN03354640	J. D. Stephens	UGA5	54	121220
	RoseaFL1_j009	SAMN03354637	J. D. Stephens	UGA16	83	105680
	RoseaAL_j010	SAMN03354638	J. D. Stephens	UGA4	51	122458
	RoseaMS_j080	SAMN03354639	J. D. Stephens	UGA7	28	127190
*S. rubra*						
	RubraGA1_j011	SAMN03354641	J. D. Stephens	UGA42	35	123685
	RubraGA2_j012	SAMN03354642	J. D. Stephens	UGA58	17	130198
	RubraGA3_j013	SAMN03354643	J. D. Stephens	UGA37	34	126171
	RubraGA4_j014	SAMN03354644	J. D. Stephens	UGA36	22	128963
	RubraGA5_j015	SAMN03354645	J. D. Stephens	UGA36	37	124041
	RubraGA6_j016	SAMN03354646	J. D. Stephens	UGA14	45	114813
	RubraSC_j017	SAMN03354661	J. D. Stephens	N/A	1	154655
	RubraSC_m001	SAMN31020178	E. Baldwin	1001	1	155181
	Rubra1_m005	SAMN31020173	E. Baldwin	1005	1	155212
	Rubra2_m006	SAMN36416360	E. Baldwin	1006	8	128073
*S. rubra ssp. gulfensis*						
	RubraGulfensisFL1_j020	SAMN03354647	J. D. Stephens	UGA3	65	115074
	RubraGulfensisFL2_j021	SAMN03354648	J. D. Stephens	UGA29	59	109430
	RubraGulfensisFL3_j022	SAMN03354649	J. D. Stephens	UGA25	23	125074
	RubraGulfensisFL1_m009	SAMN31020174	E. Baldwin	1009	1	154989
	RubraGulfensisFL2_m010	SAMN36416361	E. Baldwin	1010	9	127752
	RubraGulfensisFL3_m011	SAMN31020175	E. Baldwin	1011	1	154974
*S. rubra ssp. Rubra*						
	RubraRubraNC1_m012	SAMN31020176	E. Baldwin	1012	1	155283
	RubraRubraNC2_m013	SAMN31020177	E. Baldwin	1013	1	155302
*S. rubra ssp. viatorum*						
	RubraViatorumGA1_m014	SAMN36416362	E. Baldwin	1014	4	128950
	RubraViatorumGA2_m015	SAMN36416363	E. Baldwin	1015	13	132536
	RubraViatorumGA3_m016	SAMN31020179	E. Baldwin	1016	1	155157
	RubraViatorumGA4_m017	SAMN31020180	E. Baldwin	1017	1	155185
*S. rubra ssp. wherryi*						
	RubraWherryiAL_j027	SAMN03354650	J. D. Stephens	UGA38	38	123432
*S.leucophylla*						
	LeucophyllaFL1_j049	SAMN03354603	J. D. Stephens	UGA57	11	129896
	LeucophyllaAL1_j050	SAMN03354604	J. D. Stephens	UGA40	19	127213
	LeucophyllaGA_j051	SAMN03354605	J. D. Stephens	UGA17	19	129788
	LeucophyllaFL2_j052	SAMN03354606	J. D. Stephens	UGA56	24	132845
	LeucophyllaAL2_j053	SAMN03354607	J. D. Stephens	UGA52	12	132508
	LeucophyllaFL3_j054	SAMN03354608	J. D. Stephens	UGA6	20	126675

### 3.2 Pseudogenization of plastome encoded genes

**Figure 1:**
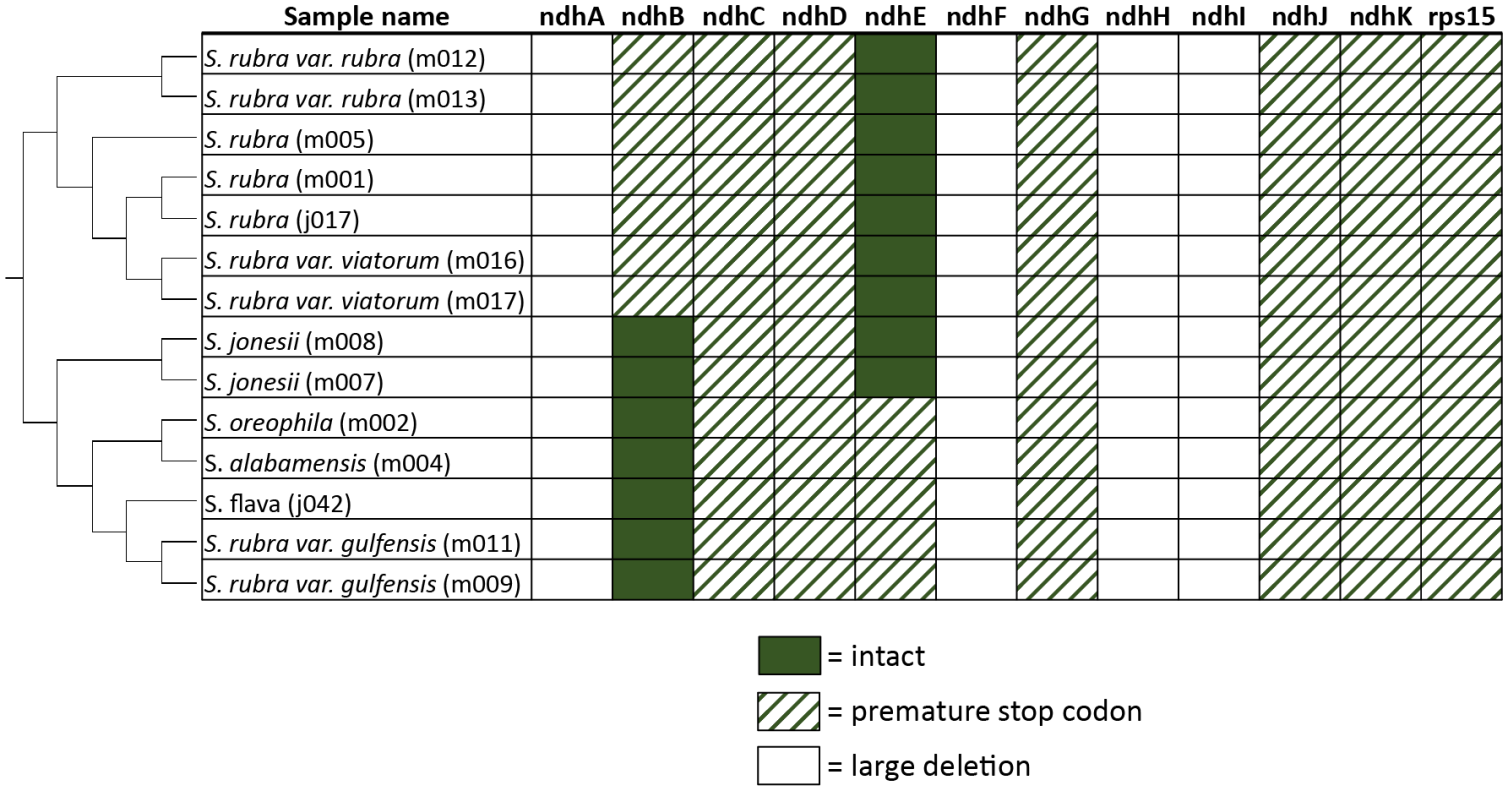
Status of *ndh* genes in all complete plastome assemblies. Filled cells represent intact genes, dashed cells represent premature stop codons, and blank cells represent genes with large deletions. Plastome tree trimmed from these samples is shown on the left.

All complete *Sarracenia* plastomes include some pseudogenized plastome-encoded genes. With the exception of *ndhB* and *ndhE*, all *ndh* genes either have been pseudogenized due to premature stop codons or large deletions (**Figure 1**). Similarly, all samples contain a premature stop codon within the *rps12* gene.

### 3.3 Plastid phylogeny

**Figure 2:**
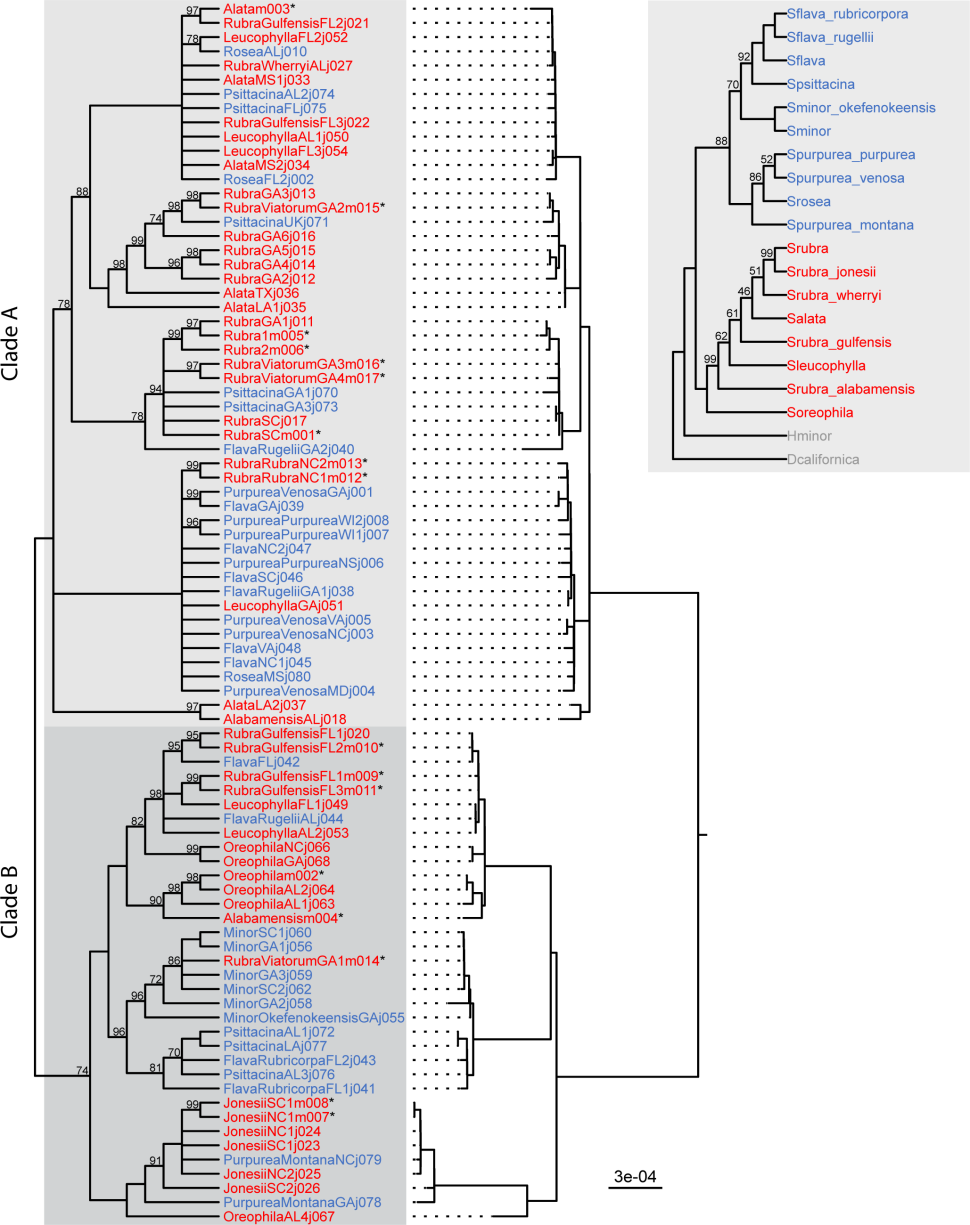
Maximum likelihood plastome cladogram (left) and phylogram (center) and species tree (inset, top right) from Stephens et al. (2015). Nodes on the cladogram with bootstrap values less than 70 are collapsed. Uncollapsed cladogram nodes with bootstrap values less than 100 are labelled. Tip names are either red or blue based on which of the two major clades the species belongs to in the species tree. Asterisks next to tip labels indicate samples that were newly sequenced for this study.

Consistent with Stephens et al. (2015), no species were found to exhibit monophyly of their plastomes, and the plastid tree is highly incongruent with the published species tree (**Figure 2**). Support values across the backbone of the tree are all greater than 70, and most internal nodes are highly supported as well (**Figure 2**). Branch lengths within *Sarracenia* are generally very short in comparison to the outgroups. An exception is the split at the base of the *Sarracenia* clade. This branch splits *Sarracenia* into two distinct plastid lineages. These main lineages are arbitrarily termed clade A and clade B (**Figure 2**). Clade B contains all sampled individuals of *minor, oreophila, jonesii*, and *purpurea* var. *montana*, and clade A contains all sampled individuals of *alata* and *purpurea* (excluding var. *montana*). All other species are split across these two main lineages (*flava, psittacina, rubra*, and *leucophylla*).

#### 3.3.1 Southern Appalachian species

**Figure 3:**
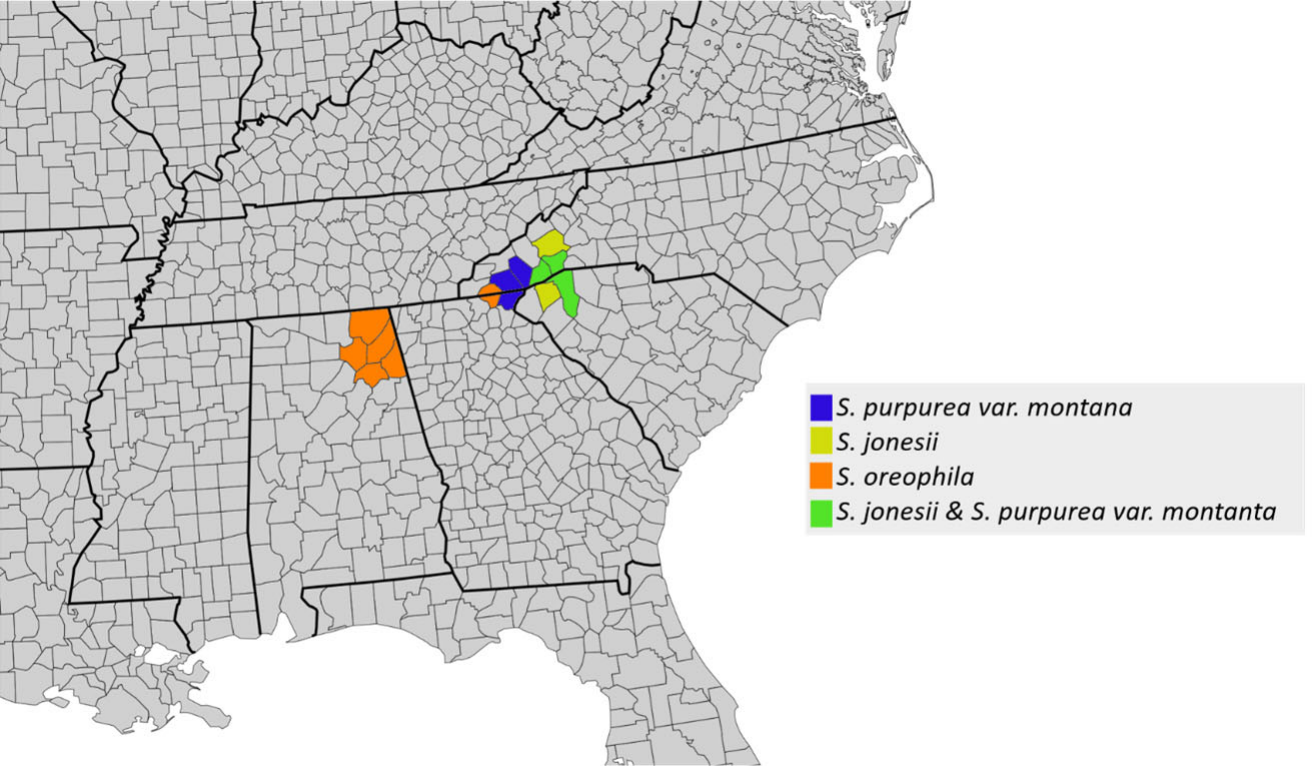
A county level distribution map for *S. purpurea var montana*, *S. jonesii*, and *S. oreophila*.

*S. purpurea* var. *montana* and *S. jonesii* form a clade. Both taxa have distributions restricted to a small area in the southern Appalachian Mountains (**Figure 3**) and hybridize at sympatric sites. The only other species found in the southern Appalachians is *S. oreophila*, although it is not sympatric with *S. jonesii* or *S. purpurea* var. *montana*, but may have been historically (McPherson and Schnell, 2011). Two *S. oreophila* accessions from Alabama are sister to the Appalachian clade, and the other *S. oreophila* accessions are placed in a clade sister to this.

#### 3.3.2 *Sarracenia flava*, *S. minor*, and *S. psittacina*

*S. flava*, *S. minor*, and *S. psittacina* form a clade sister to *S. purpurea* on the species tree, however the placement of these species on the plastid tree is not congruent. All *S. minor* accessions are placed within clade B sister to the clade containing *S. oreophila*, *S. jonesii*, and *S. purpurea* var. *montana*. Some *S. flava* and *S. psittacina* accessions from the Gulf coastal plain are also placed in the *S. minor* clade, despite all *S. minor* accessions in this study originating from the Atlantic coastal plain. This could indicate either ancient introgression or retention of plastome diversity from the ancestor of these three species. *S. flava* and *S. psittacina* are scattered across the chloroplast phylogeny; both species have accessions found in clades A and B. In *S. flava*, all Gulf coastal plain accessions are found in clade B and all Atlantic coastal plain accessions are found in clade A.

#### 3.3.3 *Sarracenia purpurea* complex

With the exception of *S. purpurea* var. *montana*, all *S. purpurea* accessions (including *S. rosea*) are placed in clade A. There is no discernible pattern to their placement within this lineage. This is surprising given the vast geographic range represented by these taxa; the individuals sampled for this study originate from throughout their distribution from Mississippi to Nova Scotia. Only *S. purpurea subsp. purpurea* is found north of Maryland, so the relatedness of plastomes between this taxon and other species are unlikely to be the result of recent introgression.

### 3.4 Plastome phylogeny simulations

**Figure 4:**
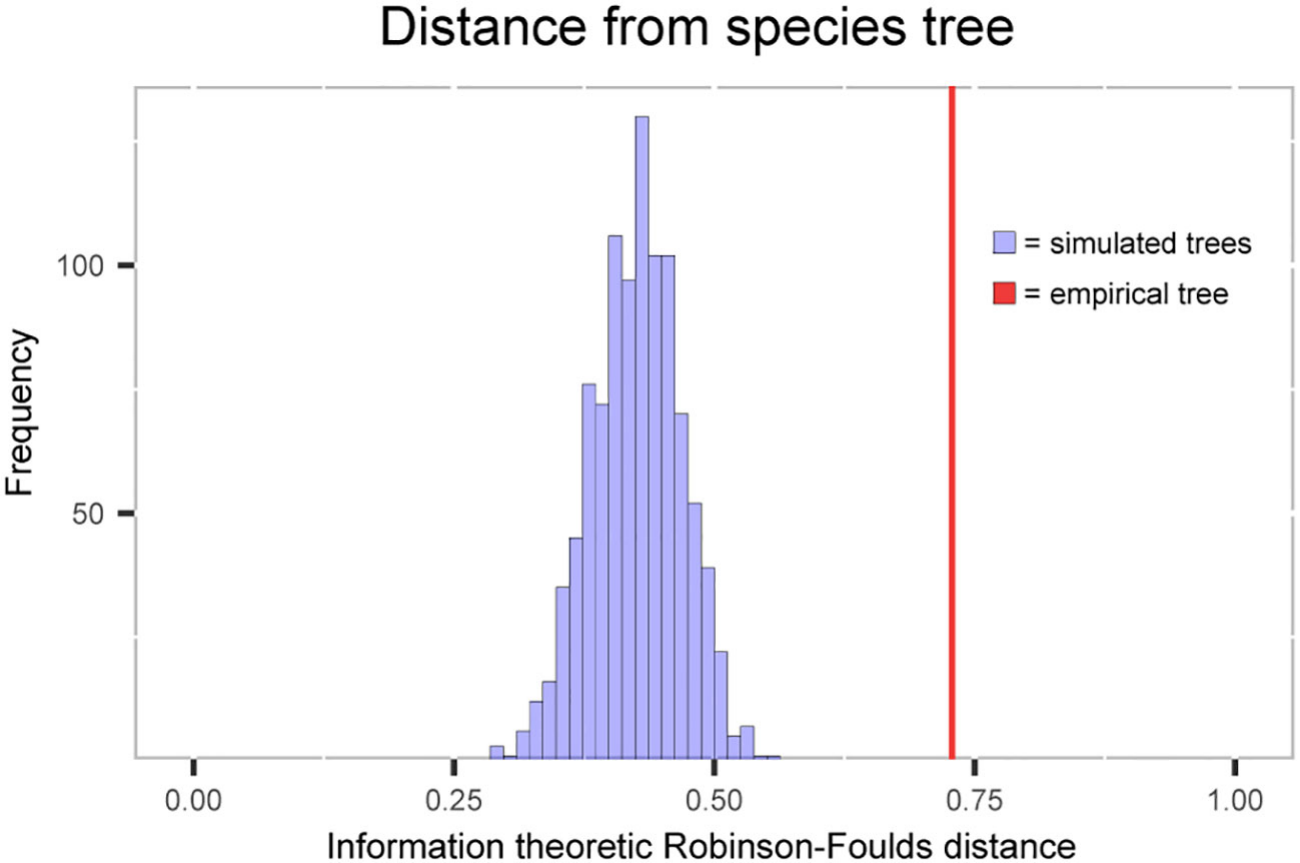
Histogram of information-based generalized Robinson-Foulds distance between the simulated plastome trees and the species tree. Red line shows the distance between empirically estimated plastome tree and the species tree.

The tree distance metric that was used ranges from 0 (an identical tree) to 1 (the most distal tree). The plastome trees simulated under the pure coalescent model have distances from the species tree ranging from 0.29 to 0.56, while the distance from the empirical plastome tree is 0.73 (**Figure 4**). A T-test using the distribution of simulated plastome tree distances as the null distribution gives a p-value of >2.2e-16, rejecting the null hypothesis of ILS causing the discordance alone.

## 4 Discussion

### 4.1 Pseudogenization of ndh genes

Independent pseudogenization or complete loss of *ndh* genes has been shown in many plant lineages, including holoparasitic, hemiparastitic, and carnivorous plant lineages (Barrett et al., 2014; Lin et al., 2017; Cao et al., 2019; Gruzdev et al., 2019; Nevill et al., 2019). Functional *ndh* genes are rarely found in non-photosynthetic parasitic plants, and the loss of *ndh* genes is strongly correlated with the transition to heterotrophy in parasitic plant lineages (Wicke et al., 2016). Since plastid encoded *ndh* genes are thought to optimize photosynthetic chemistry in fluctuating or stressful environments [reviewed in (Sabater, 2021)], the loss of *ndh* genes in parasitic lineages that are no longer fully dependent on photosynthesis as a source of carbon is unsurprising. In carnivorous plants, however, evidence for significant heterotrophic uptake of carbon is limited (Rischer et al., 2002), and a transition to full heterotrophy seems unlikely, so this line of reasoning does not explain the independent pseudogenization of functional *ndh* genes across carnivorous plant lineages. It is possible that the acquisition of organic nitrogen has an interaction with photosynthetic chemistry that relaxes the need for *ndh*. As Nevill et al. (2019) noted, organic nitrogen acquisition bypasses the need to assimilate nitrate using photosynthetically-derived reductant. Alternatively, the pseudogenization of *ndh* genes in parasitic plants and carnivorous plants could be due to unrelated mechanisms. The pseudogenization of almost all of the *ndh* genes across the genus *Sarracenia* shown here provides further evidence that carnivorous plants do not require these genes. Sequencing of full plastomes from other carnivorous species would reveal if the pseudogenization of *ndh* occurs early in carnivorous plant evolution.

### 4.2 Cytonuclear discordance

The plastome phylogeny in this study shows a similarly extreme level of discordance with the species tree as that of the Stephens et al. (2015) plastome phylogeny. That study ascribed the discordance to a combination of chloroplast capture and a lack of informative polymorphisms in the chloroplast sequence. A third source of discordance, ILS, is considered here. A lack of informative polymorphisms is not an issue here, as almost all the plastome coding sequences are used and the resulting phylogeny has high bootstrap values across the spine, suggesting that there is sufficient evidence that major clades within the tree are correct.

To distinguish between the two remaining sources of discordance, plastome phylogenies under ILS were simulated. The simulated phylogenies showed much lower levels of discordance with the species tree than the empirically estimated plastome. To simulate the plastome phylogenies, the branch lengths of the guide tree were multiplied by four due to the assumption that the chloroplast is inherited matrilineally in *Sarracenia* like most seed plants (Mogensen, 1996). Since this assumption hasn’t been empirically proven and biparental inheritance of the chloroplast is possible, simulations with branch lengths multiplied by two were performed and show similar results (**Supplemental Data**).

There is ample signal of introgression in the plastome, but Stephens et al. (2015) reported no evidence of gene flow in the nuclear data. A search through the nuclear gene trees revealed that none of the trees had a similar topology to the plastome tree, but some trees did exhibit a high degree of discordance with the species tree, possibly due to occasional nuclear gene introgression. Cytonuclear discordance is commonly observed and is attributed to introgression in plant and animal systems (Rieseberg and Soltis, 1991; Berthier et al., 2006; Gernandt et al., 2018), including several instances where there is limited signal for introgression in nuclear data (Winkler et al., 2013; Good et al., 2015; Folk et al., 2016; Rose et al., 2020). However, the mechanism for organellar introgressions without accompanying nuclear loci is poorly understood (Rieseberg and Soltis, 1991; Folk et al., 2018). *Sarracenia* is a genus where hybridization is common and thus some level of nuclear introgression might be expected. The extreme level of chloroplast capture and lack of signal for nuclear gene flow in *Sarracenia* illustrates the comparative ease of introgression of organelles over nuclear loci.

#### 4.2.1 Geographic patterns of plastome introgression

Although the lack of monophyletic species in the plastome tree makes it difficult to interpret specific instances of plastome introgression, a handful of such instances can be elucidated using geographic context. For example, all accessions of *S. purpurea* var. *montana* and *S. jonesii*, two taxa restricted to a small region in the southern Appalachians, form a well-supported clade within clade B. Given that all other *S. purpurea* accessions are placed in clade A, it is likely that a plastome derived from *S. jonesii* was introgressed into *S. purpurea* var. *montana*. Similarly, an accession of *S. rubra* that was sampled from the Georgia fall line near *S. minor* populations is placed within the *S. minor* clade. Again, we hypothesize this to be an instance of *S. minor* plastome being introgressed into *S. rubra*. More generally, the weak species clustering in the plastome tree implies a long history of interspecific exchange of cytoplasmic genomes in *Sarracenia*.

## Data availability statement

The datasets presented in this study can be found in online repositories. The names of the repository/repositories and accession number(s) can be found below: https://www.ncbi.nlm.nih.gov/bioproject/; PRJNA884359.

## Author contributions

EB, MM, JL-M contributed to study design. EB performed all data analysis and wrote initial manuscript draft. MM collected samples and generated data. MM, JL-M contributed to refinement of manuscript and approved final submission. All authors contributed to the article and approved the submitted version.

## Supplementary material

Supplementary Figure 1 png - Histograms of information-based generalized Robinson-Foulds distance between the simulated plastome trees and the species tree. Panel titles indicate how the branches of the guide tree were scaled when plastome trees were simulated. Red line shows the distance between empirically estimated plastome tree and the species tree. alignment.fasta Concatenated gene alignments used to estimate plastome tree. Plastome.newick Plastome tree in newick format. Simtrees.1.newick Plastome trees simulated with unscaled guide tree. Simtrees.2.newick Plastome trees simulated with guide tree branch lengths scaled by two. Simtrees.4.newick Plastome trees simulated with guide tree branch lengths scaled by four. Fragmented_assemblies.zip Zipped folder containing all fragmented assemblies in fasta format.
