# Supplementary figures and images for: Rampant chloroplast capture in Sarracenia revealed by plastome phylogeny
Source: Front Plant Sci. 2023 Aug 14;14:1237749. doi: 10.3389/fpls.2023.1237749 (PMC10497973; doi:10.3389/fpls.2023.1237749)

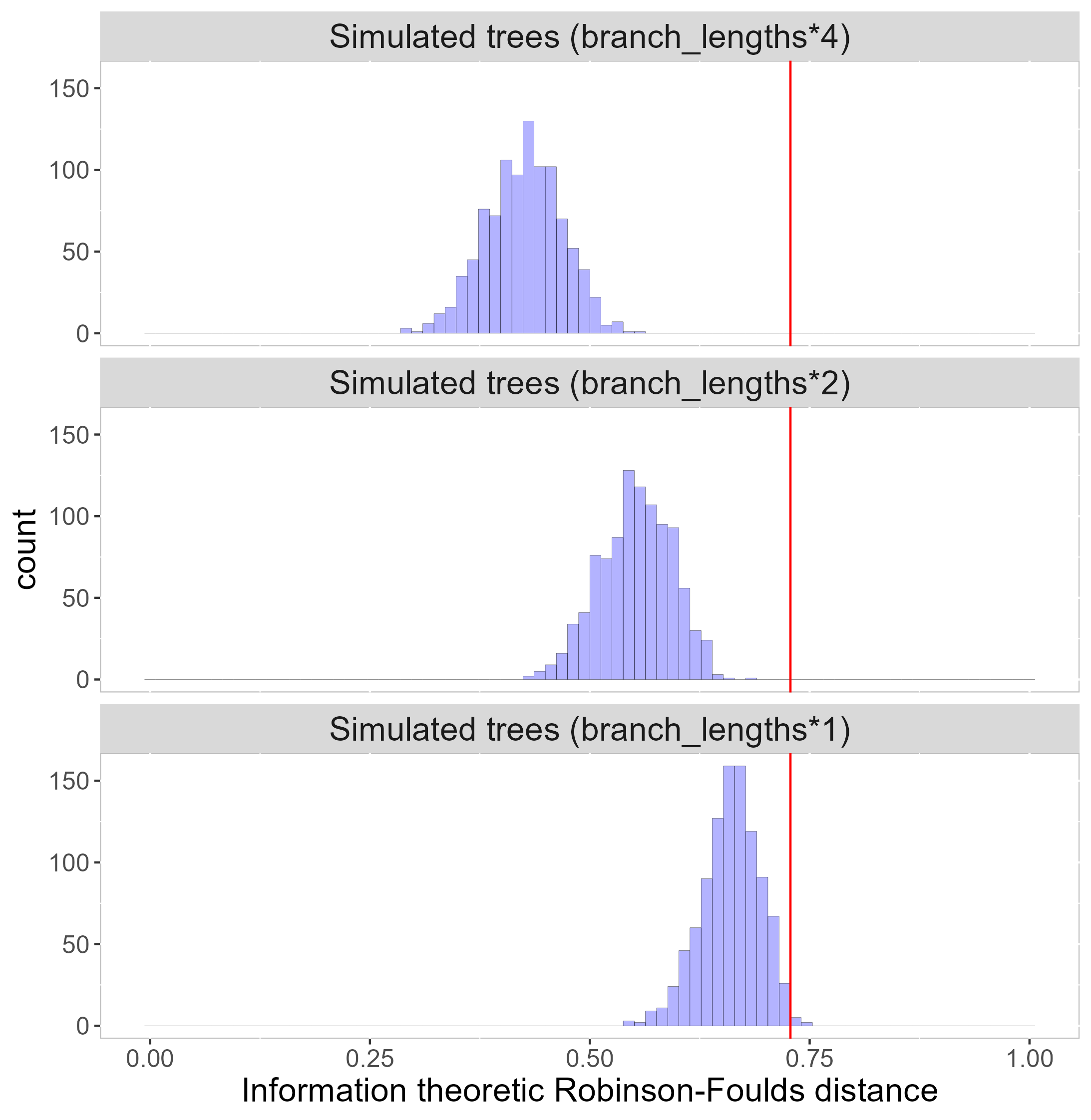

Supplement: Supplementary Figure 1 — png - Histograms of information-based generalized Robinson-Foulds distance between the simulated plastome trees and the species tree. Panel titles indicate how the branches of the guide tree were scaled when plastome trees were simulated. Red line shows the distance between empirically estimated plastome tree and the species tree. alignment.fasta Concatenated gene alignments used to estimate plastome tree. Plastome.newick Plastome tree in newick format. Simtrees.1.newick Plastome trees simulated with unscaled guide tree. Simtrees.2.newick Plastome trees simulated with guide tree branch lengths scaled by two. Simtrees.4.newick Plastome trees simulated with guide tree branch lengths scaled by four. Fragmented_assemblies.zip Zipped folder containing all fragmented assemblies in fasta format. [file DataSheet_1.zip › Supplemental_data/Supplemental_Figure_1.png]
